# Zinc Transporter ZmLAZ1-4 Modulates Zinc Homeostasis on Plasma and Vacuolar Membrane in Maize

**DOI:** 10.3389/fpls.2022.881055

**Published:** 2022-05-02

**Authors:** Bingliang Liu, Haoqiang Yu, Qinyu Yang, Lei Ding, Fuai Sun, Jingtao Qu, Wenqi Feng, Qingqing Yang, Wanchen Li, Fengling Fu

**Affiliations:** Key Laboratory of Biology and Genetic Improvement of Maize in Southwest Region, Ministry of Agriculture, Maize Research Institute, Sichuan Agricultural University, Chengdu, China

**Keywords:** maize, ZmLAZ1-4, zinc transport, tonoplast, transcriptional regulation

## Abstract

Zinc is an essential micronutrient for plant growth and development, and functions as a cofactor for hundreds of transcription factors and enzymes in numerous biological processes. Zinc deficiency is common abiotic stress resulting in yield loss and quality deterioration of crops, but zinc excess causes toxicity for biological systems. In plants, zinc homeostasis is tightly modulated by zinc transporters and binding compounds that uptake/release, transport, localize, and store zinc, as well as their upstream regulators. Lazarus 1 (LAZ1), a member of DUF300 protein family, functions as transmembrane organic solute transporter in vertebrates. However, the function of LAZ1 in plants is still obscure. In the present study, the ZmLAZ1-4 protein was confirmed to bind to zinc ions by bioinformatic prediction and thermal shift assay. Heterologous expression of *ZmLAZ1-4* in the zinc-sensitive yeast mutant, *Arabidopsis*, and maize significantly facilitated the accumulation of Zn^2+^ in transgenic lines, respectively. The result of subcellular localization exhibited that ZmLAZ1-4 was localized on the plasma and vacuolar membrane, as well as chloroplast. Moreover, the *ZmLAZ1-4* gene was negatively co-expressed with *ZmBES1/BZR1-11* gene through co-expression and real-time quantitative PCR analysis. The results of yeast one-hybrid and dual-luciferase assay suggested that ZmBES1/BZR1-11 could bind to *ZmLAZ1-4* promoter to inhibit its transcription. All results indicated that ZmLAZ1-4 was a novel zinc transporter on plasma and vacuolar membrane, and transported zinc under negative regulation of the ZmBES1/BZR1-11 transcription factor. The study provides insights into further underlying the mechanism of ZmLAZ1-4 regulating zinc homeostasis.

## Introduction

Zinc (Zn) is an essential micronutrient for plant growth and development, and functions as a cofactor for hundreds of transcription factors and enzymes in numerous biological processes, such as chlorophyll biosynthesis, gene expression, signal transduction, and stress response (Palmer and Guerinot, 2009; Zlobin, 2021). Zn deficiency is common abiotic stress resulting in production loss and quality deterioration of crops, but Zn excess causes toxicity for biological systems (Grotz et al., 1998; Arrivault et al., 2006; Palmer and Guerinot, 2009). However, Zn deficiency is far more frequent than toxicity, because Zn content in the soils has low availability for plants (Alvarez and Rico, 2003; Cakmak, 2008). Zn toxicity only occurs on polluted soils containing excessive Zn in mining or industrial areas (Mossa et al., 2020). In plants, the excessive Zn is usually stored in the vacuole to avoid toxicity (Martinoia et al., 2007). Usually, Zn homeostasis is tightly modulated by Zn transporters and binding compounds that uptake/release, transport, localize, and store Zn within the whole plant as well as within individual tissues, cells, and cellular compartments (Palmer and Guerinot, 2009; Zlobin, 2021). Some metal tolerance proteins (MTPs) and heavy metal ATPases (HMAs) localize on the vacuolar membrane and modulate Zn homeostasis as a Zn sensor and transporter (Arrivault et al., 2006; Lan et al., 2013; Menguer et al., 2013; Tanaka et al., 2015).

Zn uptake from the soil, as well as transport in organs, tissues, cells, and intracellular compartments, is mediated by some members of the zinc–iron permease (ZIP) family on plasma and vacuolar membrane (Grotz et al., 1998; Milner et al., 2013). Overexpression of *ZIP* genes is responsive to Zn deficiency and restores Zn uptake in yeast mutants (Ishimaru et al., 2007; Evens et al., 2017; Yang et al., 2020). In addition, the Zn-regulated transporter (ZRT), iron-regulated transporter (IRT), and natural resistance-associated macrophage protein (NRAMP) have been reported to uptake and transport Zn (Wang et al., 2021). Furthermore, the expression of these Zn transporters is negatively regulated by upstream transcription factors such as members of the bZIP family, which directly bind to zinc deficiency response elements in the promoters of *ZIP* and other Zn transporter genes (Evens et al., 2017; Nazri et al., 2017; Pita-Barbosa et al., 2019; Lilay et al., 2021). Zinc in the cytoplasm is trapped by small cysteine-rich proteins (metallothioneins) and cysteine-containing peptides (phytochelatins). Consequently, the concentration of free Zn is kept at a low level in cytoplasm, thus protecting cells against Zn toxicity (Cobbett, 2000). The plasma membrane of plant cells contains at least two Zn extrusion transporters including HMA2 and HMA4 (Chong et al., 2009). In *Arabidopsis*, these ATPases release excessive Zn in the cytosol and mediate the intercellular and intertissue Zn transport (Hussain et al., 2004). ZIF1 (zinc-induced facilitator-1) also acts as a Zn transporter (Haydon and Cobbett, 2007). Zinc is also required in the chloroplast as cofactors for superoxide dismutase, which catalyze the conversion of superoxide to hydrogen peroxide, preventing cellular damage by the reactive hydroxyl radical species (Palmer and Guerinot, 2009). A possible candidate of Zn transporter across the chloroplast membrane is HMA1, which localizes to the chloroplast membrane and contributes to the detoxification of Zn excess (Moreno et al., 2008; Kim et al., 2009; Mikkelsen et al., 2012).

Maize is much more sensitive to Zn deficiency than other crops (Alvarez and Rico, 2003; Mattiello et al., 2015). So, Zn deficiency is recognized as one of the main limiting factors for maize yield. The application of Zn fertilizer achieves a yield gain of more than 18% (Potarzycki and Grzebisz, 2009; Xue et al., 2014; Hacisalihoglu, 2020). In maize, the expression of some *ZmZIP* genes is significantly increased under Zn deficiency (Mondal et al., 2014; Khatun et al., 2018; Mager et al., 2018). Likewise, overexpression of *ZmZIP3*, *5*, *7*, and *ZmIRT1* genes increases Zn accumulation in transgenic maize and *Arabidopsis* (Li et al., 2015, 2016, 2019). Besides, little is known about other genes in the Zn regulation of maize.

Lazarus 1 (LAZ1) is a transmembrane protein with sequence homology and structural similarity to members of the DUF300 family (Malinovsky et al., 2010). DUF300 proteins function as transmembrane organic solute transporter in vertebrates (Wang et al., 2001). In *Arabidopsis*, two LAZ1 proteins are found to maintain vacuole integrity and mediate brassinosteroid (BR) signaling and localized on the vacuolar membrane (Liu et al., 2018). In our previous study, we cloned eight members of the *ZmLAZ1* gene family from maize and found their differential expression among different organs, developmental stages, and under abiotic stresses, implying their functional diversity (Liu et al., 2020). In the present study, we demonstrated that the ZmLAZ1-4 protein was distinct from the other seven members, and it functioned as a zinc transporter on plasma and vacuolar membrane and modulated zinc homeostasis under the negative regulation of BR signaling transcription factor ZmBES1/BZR1-11.

## Materials and Methods

### Substrate Prediction and Thermal Shift Assay

To predict candidate substrates of ZmLAZ1 proteins, the amino acid sequences of eight ZmLAZ1 members were submitted to the SWISS-MODEL software^1^ to get a protein model structure file in PDB format. Then the PDB file of each ZmLAZ1 was searched against the RCSBPDB software^2^ to get putative substrates. The coding sequences (CDSs) of the *ZmLAZ1-4* and *ZmLAZ1-8* genes were amplified from pMD19-T-*ZmLAZ1-4* and pMD19-T-*ZmLAZ1-8* (Liu et al., 2020) by PCR primers CDS1-4F/CDS1-4R and CDS1-8F/CDS1-8R (Supplementary Table 1), respectively, and inserted into His-tagged prokaryotic expression vector pET-32a (Takara, Osaka, Japan) by using ClonExpress II One Step Cloning Kit (Vazyme Biotech, Nanjing, China). The construct was introduced into *Escherichia coils* BL21 (DE3), screened on Luria-Bertani (LB) plates containing 100 mg/ml ampicillin, and grown in LB medium at 37°C to OD_600_ = 0.6. The His-tagged proteins were induced by 0.1 mM isopropyl β-D-1-thiogalactopyranoside (IPTG) at 16°C overnight, purified by using Ni-TED 1 ml Sefinose (TM) Column (Sangon Biotech, Shanghai, China), detected by 10% sodium dodecyl sulfate-polyacrylamide gel electrophoresis (SDS-PAGE), quantified in NanoDrop One Microvolume UV–Vis Spectrophotometer (Thermo Fisher Scientific, Waltham, MA, United States), and diluted to 1 μg/μl with 10% dimethyl sulfoxide (DMSO).

As described by Huynh and Partch (2015) with minor modification, 2 μl of 10% DMSO, 6 μg of the purified protein, 2 μl of 10 × SYPRO orange, and 0 (blank control) and 200 μM of each predicted substrate were added into each of three wells of a 96-well PCR plate. In CFX Connect Real-Time PCR Detection System (Bio-Rad, Hercules, CA, United States), the sampled plate was equilibrated at 25°C for 5 min and then ramped up to a final temperature of 95°C in increments of 1°C. Fluorescence was read every 0.2°C ramping up. The change rates of relative fluorescence units (RFUs) with time (T) [-d(RFU)/dT] were plotted vs. the temperature to generate melting curves of ZmLA1-4 incubated with each predicted substrate.

### Zinc Transport Assay in Zinc-Sensitive Yeast Mutant

The CDS of *ZmLAZ1-4* was amplified from pMD19-T-*ZmLAZ1-4* plasmid (Liu et al., 2020) by using primers pYES2F/pYES2R (Supplementary Table 1) and used for construction of yeast expression vector pYES2-*ZmLAZ1-4* by using ClonExpress^®^ II One Step Cloning Kit (Vazyme Biotech, Nanjing, China). The pYES2-*ZmLAZ1-4* and empty vector pYES2 were transformed into yeast Zn-sensitive mutant Δ*zrc1* (Miyabe et al., 2001) and wild-type (WT) strain BY4743, respectively. The positively transformed lines were selected on synthetic dropout medium (SD) plates containing 2% galactose (w/v) and without uracil (Ura), identified by PCR amplification with primers LAZ4F/LAZ4R (Supplementary Table 1), grown in SD medium at 30°C for 16 h, diluted to OD_600_ = 0.8, and then diluted by 10-fold serial to 1:10^4^. Subsequently, 5 μl of each dilution were spotted on SD plates containing ZnSO_4_ (0 or 2 mM) and 2% galactose with three replicates and incubated at 30°C for 3 days. Meanwhile, 50 μl transformed lines were grown in a 10-ml SD liquid medium containing ZnSO_4_ (1 and 2 mM) and 2% galactose, and used for measurement OD_600_ at 0, 6, and 24 h. Then the cultures were washed with 10 μM ethylene diamine tetraacetic acid (EDTA) and used for the determination of Zn concentration by Inductively Coupled Plasma-Mass Spectrometry (Thermo Fisher Scientific, Waltham, MA, United States).

### Transformation and Phenotyping of *Arabidopsis* and Maize

Overexpression vector (pC2300-*35S*-*ZmLAZ1-4*) of *ZmLAZ1-4* was constructed as above and introduced into *Agrobacterium tumefaciens* strain GV3101. Positive strains were identified and used for transformation of WT *Arabidopsis thaliana* by floral dip. Transgenic lines were screened on kanamycin 1/2 MS plates and identified by PCR with primers LAZ4F1/LAZ4R1 (Supplementary Table 1). Referring to Kawachi et al. (2009), each homozygous line was grouped into three replicates and grown on 1/2 MS zinc deficiency plates (control) with 5 and 50 μM ZnSO_4_ at 22°C temperature, 50% humidity, and 16 h light of 120 μE m^–2^ s^–1^ illumination intensity/8 h dark period for 2 weeks. After photographing for the phenotype, the seedlings were dried at 60°C for 48 h, weighed for biomass, and digested in 80% nitric acid at 250°C overnight. The digested solution was diluted with ddH_2_O and used for measurement of Zn^2+^ content by Inductively Coupled Plasma-Mass Spectrometry (Thermo Fisher Scientific, Waltham, MA, United States).

Overexpression vector (pZZ00026-*Ubi-ZmLAZ1-4-Tnos*) of *ZmLAZ1-4* was constructed as above and used to transform embryonic calli isolated from maize inbred line B73 by *Agrobacterium* mediation. Positive calli were screened on H6 medium with 0.06% (v/v, effective concentration) Basta herbicide. Regenerated plantlets were screened by PAT/bar EPSPS LFD Strip kit (Youlong, Shanghai, China) according to the manufacturer’s instruction and identified by PCR with primers LAZ4F2/LAZ4R2 (Supplementary Table 1) and real-time quantitative PCR (RT-qPCR) with primers LAZ4F3/LAZ4R3 (Supplementary Table 1). Homozygous T_3_ lines and WT were grown in vermiculite with Zn dropout Hoagland’s nutrient solution (Coolaber, Beijing, China) at 28°C and 300 μE m^–2^ s^–1^ illumination intensity for 16 h/20°C dark for 8 h. Referring to Kawachi et al. (2009), at three-leaf stage, the seedlings of each line were grouped into three replicates, treated with 5 and 50 μM ZnSO_4_ for 3 weeks, then photographed and dried at 60°C for 72 h, and weighed for biomass and used to measure Zn^2+^ content as above.

### Subcellular Localization

The transmembrane domains of LAZ1-4 were predicted by the TMHMM v. 2.0 software.^3^ The CDS without termination codons of *ZmLAZ1-4* and tonoplast maker gene *AtTIP2* (Loque et al., 2005) was amplified from pMD19-T-*ZmLAZ1-4* plasmid (Liu et al., 2020) and *Arabidopsis* cDNA using primers Non-Term1-4F/Non-Term1-4R and AtTIP2F/AtTIP2R (Supplementary Table 1) and used for construction of transient expression vector *35S-ZmLAZ1-4-eGFP* and *35S-mCherry-AtTIP2* using ClonExpress^®^ II One Step Cloning Kit (Vazyme Biotech, Nanjing, China), respectively. Subsequently, the *35S-ZmLAZ1-4-eGFP*, *35S-mCherry-AtTIP2* of tonoplast maker, *35S-mCherry-OsRac3* of plasma membrane marker (donated by professor Shuangcheng Li, Tao et al., 2021), and empty vector *35S-eGFP* (blank control) were introduced into *Agrobacterium tumefaciens* strain GV3101, respectively.

Maize mesophyll protoplasts were prepared with etiolated leaves and co-transfected with *35S-ZmLAZ1-4-eGFP* and *35S-mCherry-OsRac3*, as well as *35S-eGFP* and *35S-mCherry-OsRac3* as blank control. After incubation at 25°C in dark for 12 h, the protoplasts were used to observe the fluorescence of eGFP and OsRac3 under laser scanning confocal microscope LSM 800 with 488 and 584 nm laser channel (Zeiss, Oberkochen, Germany), respectively.

The combination of *35S-ZmLAZ1-4-eGFP* and *35S-mCherry-OsRac3*, *35S-ZmLAZ1-4-eGFP* and *35S-mCherry-AtTIP2*, as well as *35S-eGFP* and *35S-mCherry-OsRac3*, and *35S-eGFP* and *35S-mCherry-AtTIP2* plasmid was mixed with 0.1 M spermidine and 2.5 M CaCl_2_ and precipitated onto gold particles (φ = 60 μm), respectively. Onion bulbs were surface sterilized with 75% ethanol. The fifth scales without pigment were cut into 2 cm × 2 cm, incubated on MS medium for 4 h, and bombard in helium biolistic gun (Bio-Rad, Hercules, CA, United States) with above gold particles. After filtration at 28°C under dark for 24 h, the bombarded onion scales were used to observe the fluorescence of eGFP, OsRac3, and AtTIP2 under the same microscope.

The *Agrobacterium* strains harboring *35S-ZmLAZ1-4-eGFP* and *35S-eGFP* were infiltrated into the abaxial leaf surface of 3-week-old plants of *Nicotiana benthamiana*, respectively. After incubation at 22°C and 14 light/10 dark for 24 h, the infiltrated leaves were used to observe the fluorescence of eGFP and autofluorescence of chloroplasts under the same microscope.

### Co-expression and Real-Time Quantitative PCR Analysis

The transcriptomic data of maize inbred line B73 were downloaded from MazieGDB^4^ and used for co-expression analysis with *ZmLAZ1-4* by using a Perl script (Supplementary Data Set 1). The correlation coefficient was set as > 0.9 and < −0.9. Among the candidates, only the *ZmBES1/BZR1-11* gene encoded transcription factor, co-expressed with *ZmLAZ1-4* (correlation coefficient is −0.93), and used for RT-qPCR analysis. The seeds of inbred line B73 were surface sterilized with 30% H_2_O_2_, germinated in petri dish, and transplanted into a plastic mesh grid for hydroponic culture at 28°C under a photoperiod of 14 h light/10 h dark. At the three-leaf stage, the seedlings were subjected to the treatment of 5 μM ZnSO_4_. At the 0 (control), 1st, 2rd, and 3rd day of treatment, the whole plant was sampled, ground in liquid nitrogen, and used for total RNA extraction by using RNAiso plus kit (TaKaRa, Osaka, Japan). After removing probable genomic DNA contamination by using RNase-free DNase (TaKaRa, Osaka, Japan), these samples were quantified on NanoDrop 2000 (Thermo Fisher Scientific, Waltham, MA, United States) and reverse transcribed into cDNA by using PrimeScript™ reagent kit (TaKaRa, Osaka, Japan). The RT-qPCR was performed as described by Sun et al. (2020). The *ZmGAPDH* was used as reference. The primers are listed in Supplementary Table 1.

### Yeast One Hybrid and Dual Luciferase Assay

The CDS of *ZmBES1/BZR1-11* was amplified with primers pGADT7F/pGADT7R (Supplementary Table 1) and used to construct vector pGADT7-*ZmBES1/BZR1-11*. The *cis-*acting elements bound by ZmBES1/BZR1-11 in *ZmLAZ1-4* promoter were predicted by PlantCARE.^5^ The sequence (−1 to −1,100 bp) of *ZmLAZ1-4* promoter (*pZmLAZ1-4*) containing *cis-*acting elements was amplified with primers pAbAiF/pAbAiR (Supplementary Table 1) and used to construct reporter vector pAbAi-*pZmLAZ1-4*. The pAbAi-*pZmLAZ1-4* was restricted with *Bbs*I and transformed into yeast Y1H gold by using a yeast transformation kit (Coolaber, Beijing, China). The transformant was plated onto Ura dropout SD medium and incubated at 30°C for 5 days. The positive clones were identified by PCR across the multiple cloning sites of the pAbAi vector and the *ura3-52* gene of Y1H gold with primers Y1HF/Y1HR (Supplementary Table 1), diluted to 10^–1^, 10^–2^, 10^–3^, and 10^–4^ folds with 0.9% NaCl and plated onto Ura dropout SD medium containing either 50, 100, 200, or 400 ng/ml aureobasidin (AbA) to inhibit the Y1H gold background. Competent cells were prepared with the positive clones, transformed with prey vector pGADT7-*ZmBES1/BZR1-11*, plated onto the Leu dropout SD medium containing AbA at an optimal concentration, and incubated at 30°C for 5 days.

The promotor sequence (−1 to −1,100 bp) of *ZmLAZ1-4* (*pZmLAZ1-4*) was amplified with specific primers pGreenIIF/pGreenIIR (Supplementary Table 1) and inserted into pGreenII-0800*-LUC* plasmid to drive firefly luciferase gene (*LUC*) and generate reporter vector *pZmLAZ1-4-LUC*. The *Renilla* luciferase gene *REN* driven by *35S* promoter in *pZmLAZ1-4-LUC* plasmid was used as internal reference. The CDS of the *ZmBES1/BZR1-11* gene was amplified with primers pCAMBIA2300F/pCAMBIA2300R (Supplementary Table 1) and inserted into pCAMBIA2300-*35S*-eGFP plasmid to create effector vector *35S*-*ZmBES1/BZR1-11*. The reporter and the effector vectors were introduced into *Agrobacterium* strain GV3101, respectively, and used for co-infiltration of *Nicotiana benthamiana* leaves. After incubated at 22°C and 14 light/10 dark for 3 days, the leaves were visualized for LUC signal in ChemiDoc™ Imaging System (Bio-Rad, Hercules, CA, United States). The relative activities of LUC and REN were determined in a dual-luciferase reporter assay system (Thermo Fisher Scientific, Waltham, MA, United States) and used to calculate relative LUC activity (LUC/REN).

### Statistical Analysis

All experiments were performed with three replicates. The data were shown as mean ± standard deviation and analyzed using Student’s *t*-test at *p < 0.05 and **p < 0.01 level.

## Results

### ZmLAZ1-4 Specifically Binds to Zinc

By the RCSBPDB software as described by Wang et al. (2001), substrates of eight ZmLAZ1 members were mainly predicted to be organic solutes including α-D-mannose, β-D-mannose, N-acetyl-D-glucosamine, cholesterol, phosphocholine, ethanesulfonic acid, phosphinic acid, toporphyrin, and octyl-β-octylglucoside. However, only ZmLAZ1-4 (Zm00001d012921) and ZmLAZ1-8 (Zm00001d036361) were predicted to combine inorganic ions containing zinc (Zn^2+^), magnesium (Mg^2+^), and calcium (Ca^2+^) (Table 1). During many times of prokaryotic expression, the ZmLAZ1-8 protein was not successfully purified (Supplementary Figure 1). Therefore, the ZmLAZ1-4 was used for further study.

**TABLE 1:** Predicted substrates for the ZmLAZ1 family.

Protein	Substrate					
ZmLAZ1-1	–					
ZmLAZ1-2	MAN	BMA	NAG	CE		
ZmLAZ1-3	–					
ZmLAZ1-4	Zn^2+^	MAN	BMA	Mg^2+^	NAG	OPA
ZmLAZ1-5	MAN	BMA	NAG	CE		
ZmLAZ1-7	Dodecane	Retinal	Decane	PPC		
ZmLAZ1-8	ESA	Zn^2+^	Enoate	Ca^2+^	TPP + Fe	OBG
ZmLAZ1-9	–					

*MAN, α-D-mannose; BMA, β-D-mannose; NAG, N-acetyl-D-glucosamine; CE, Cholesterol; PPC, Phosphocholine; ESA, ethanesulfonic acid; OPA, Oxyphosphinic acid; TPP, toporphyrin; OBG, octyl-β-octylglucoside—means no predicted substrate.*

**FIGURE 1:**
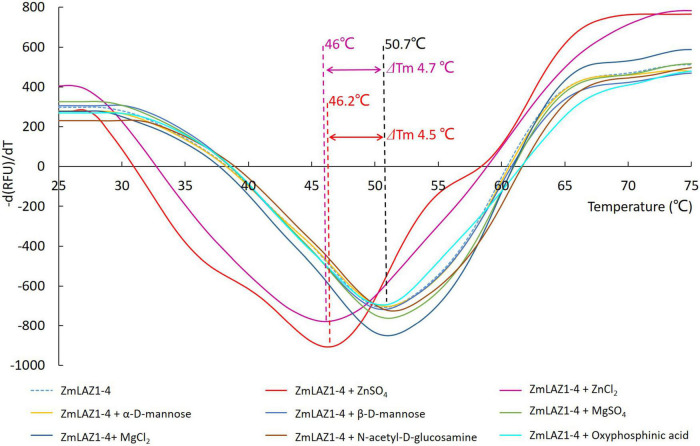
Thermal shift assay of ZmLA1-4 incubated with predicted substrates.

In the thermal shift assay, melting temperature (Tm) of the ZmLAZ1-4 protein incubated with ZnCl_2_ and ZnSO_4_ was 4.7 and 4.5°C lower than that of ZmLAZ1-4 incubated alone (blank control), respectively, whereas Tm of ZmLAZ1-4 incubated with other predicted substrates kept same value with blank control (Figure 1). This result suggested that zinc ion was candidate substrate of ZmLAZ1-4.

### ZmLAZ1-4 Transports Zinc in Yeast, *Arabidopsis*, and Maize

**FIGURE 2:**
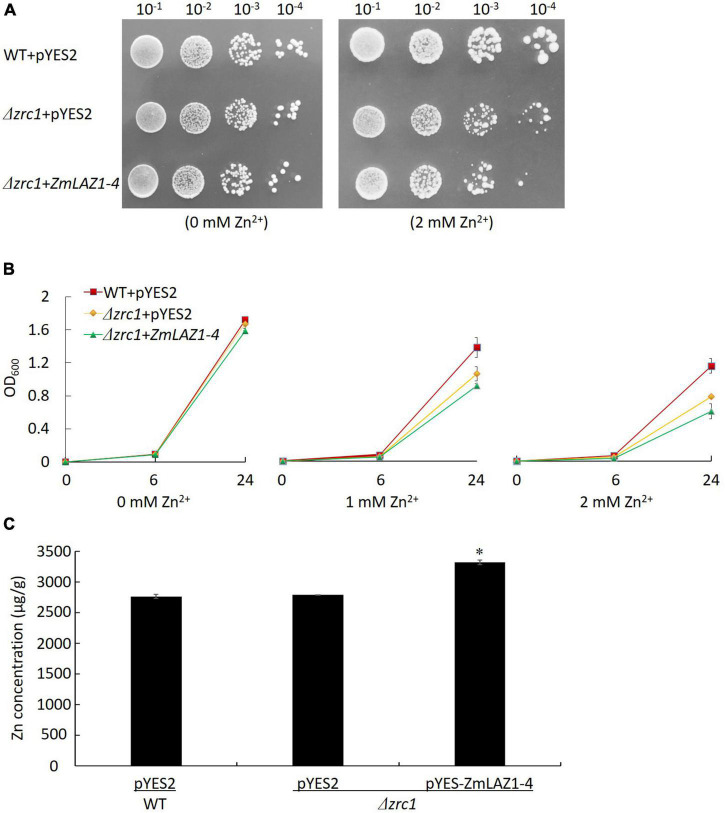
Zinc transport assay in yeast Zn-sensitive mutant. **(A)** Colonies of WT and Δ*zrc1* mutant transformed by empty vector and Δ*zrc1* transformed by *ZmLA1-4* under 0 and 2 mM ZnSO_4_ stress. **(B)** OD_600_ curves of the three lines under 0, 1, and 2 mM ZnSO_4_ stress. **(C)** Zn concentration in yeast grown in synthetic dropout medium (SD) with 2 mM ZnSO_4_ for 48 h. *p < 0.05.

Under non-stress (0 mM Zn^2+^), the diluted colonies and growth curves of OD_600_ showed no significant difference among Δ*zrc1* mutant transformed by the *ZmLAZ1-4* gene, and Δ*zrc1* and WT transformed by empty vector pYES2. Under 2 mM Zn^2+^ stress, the difference was significant among these three lines (Figures 2A,B). The complementation of *ZmLAZ1-4* significantly inhibited the growth of Zn-sensitive mutant Δ*zrc1*. The Zn^2+^ concentration of Δ*zrc1* transformed by *ZmLAZ1-4* was significantly higher than that of Δ*zrc1* and WT transformed by empty vector pYES2 (Figure 2C), suggesting that ZmLAZ1-4 could transport Zn^2+^ into cells.

**FIGURE 3:**
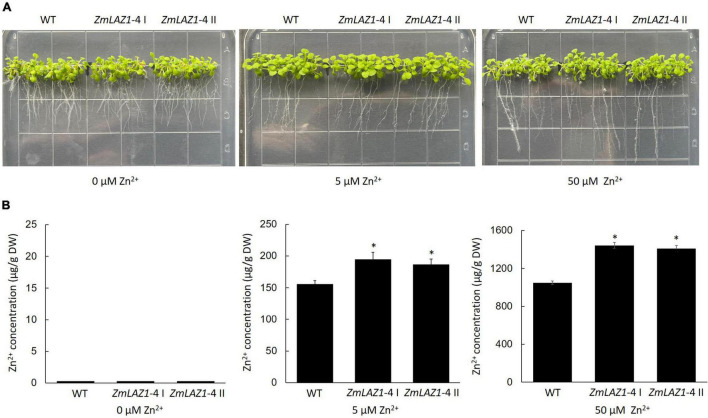
Phenotype and Zn^2+^ content of transgenic *Arabidopsis*. **(A)** Phenotype. **(B)** Zn^2+^ content. The seeds of every line were sterilized and grown on 1/2 MS zinc deficiency plates (control) with 5 and 50 μM ZnSO_4_ for 2 weeks, respectively. WT, wild type. *ZmLA1-4* I and *ZmLA1-4* II represent homozygous lines. *p < 0.05.

Two homozygous T_3_ *Arabidopsis* lines overexpressing *ZmLAZ1-4* were screened on kanamycin 1/2 MS plates and identified by PCR amplification (Supplementary Figure 2). Under 0, 5, and 50 μM ZnSO_4_ treatments, the growth phenotype of T_3_ lines showed no obvious difference compared to WT (Figure 3A). However, Zn^2+^ content of transgenic lines was significantly higher than WT under 5 and 50 μM ZnSO_4_ treatments, while only trace content was measured under 0 μM ZnSO_4_ treatment (Figure 3B).

**FIGURE 4:**
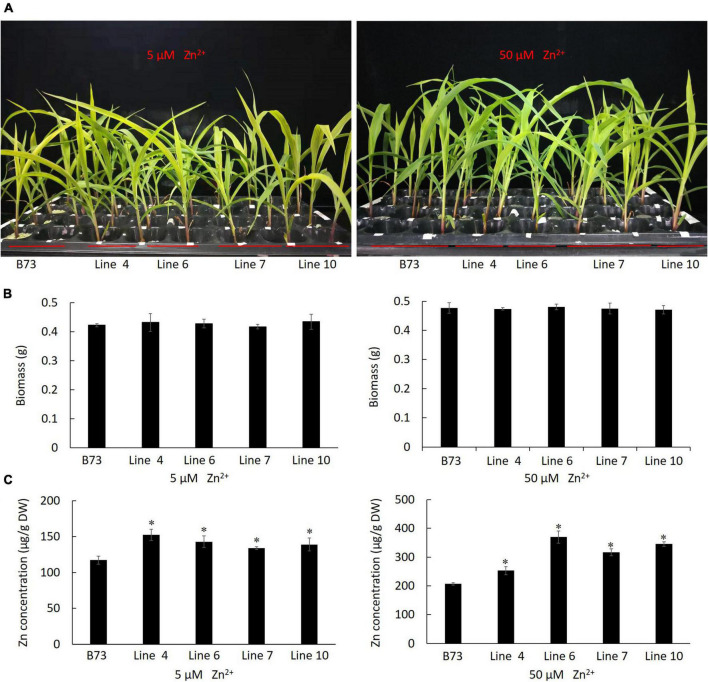
Phenotype of transgenic maize under ZnSO_4_ treatment. **(A)** Phenotype. **(B)** Biomass. **(C)** Zn^2+^ content. At three-leaf stage, the seedlings of each line were grouped into three replicates, treated with 5 and 50 μM ZnSO_4_ for 3 weeks, then photographed and dried at 60°C for 72 h, and weighed for biomass and used to measure Zn^2+^ content. The biomass of three seedlings of every line was shown. B73, the untransformed control. Line 4, 6, 7, and 10 are homozygous T_3_ lines. *p < 0.05.

By *A. tumefaciens*-mediated embryonic calli transformation, from the positive transgenic calli harboring *ZmLAZ1-4*, ten plantlets were regenerated and four homozygous T_3_ maize lines overexpressing *ZmLAZ1-4* were identified by PAT/bar EPSPS LFD Strips (Supplementary Figure 3), PCR amplification (Supplementary Figure 4), and RT-qPCR (Supplementary Figure 5). Four homozygous lines and WT were grown in vermiculite with Zn deficient Hoagland’s nutrient solution. After 3 weeks of 5 and 50 μM ZnSO_4_ treatments, the growth phenotype and biomass of all transgenic lines showed different compared with WT (Figures 4A,B). However, the Zn^2+^ content of all transgenic lines was significantly higher than WT under 5 and 50 μM ZnSO_4_ treatments (Figure 4C). The above results indicated that ZmLAZ1-4 functioned as a Zn transporter.

### ZmLAZ1-4 Localized on Plasma and Vacuolar Membranes

**FIGURE 5:**
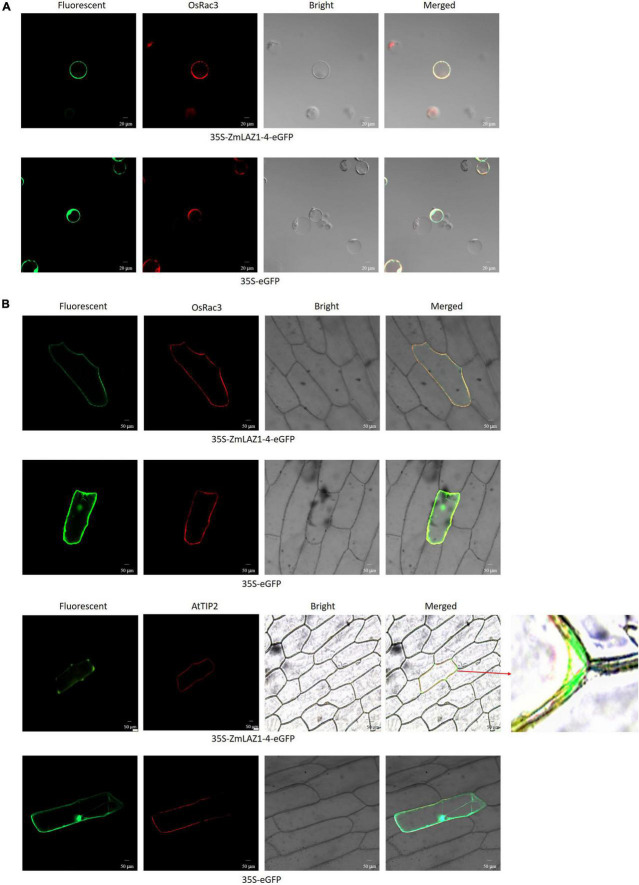
Subcellular localization of ZmLAZ1-4 in maize protoplasts **(A)** and onion cells **(B)**. Scale bar is 50 μm.

By the TMHMM software, seven transmembrane domains were predicted during ZmLA1-4 protein (Supplementary Figure 6). As shown in Figure 5, the GFP fluorescence was observed in the cytoplasm and nucleus in maize protoplasts, and onion cells transfected by empty vector *35S-eGFP*. However, the GFP fluorescence from the fusion protein (35S-ZmLAZ1-4-GFP) was merged with red fluorescence of the plasma membrane marker OsRac3, tonoplast maker AtTIP2, and autofluorescence of chloroplasts. Especially when AtTIP2 was used as a maker, it could be clearly seen that ZmLAZ-4 was localized on the tonoplast. Furthermore, the ZmLAZ1-4 was also localized to chloroplast (Supplementary Figure 7). These results indicated the subcellular localization of the ZmLAZ1-4 protein on the plasma and vacuolar membrane.

### ZmLAZ1-4 Is Negatively Regulated by ZmBES1/BZR1-11

**FIGURE 6:**
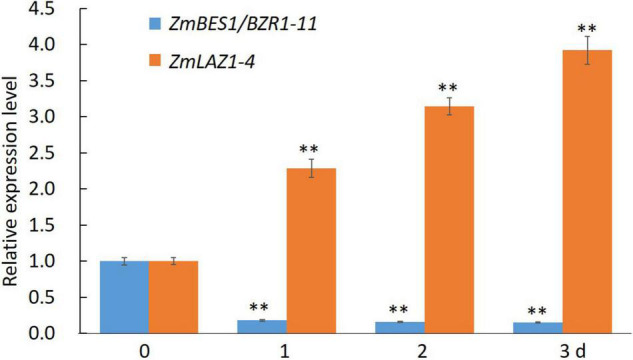
Relative expression level of *ZmLAZ1-4* and *ZmBES1/BZR1-11* in response to Zn deficiency. **p < 0.01.

**FIGURE 7:**
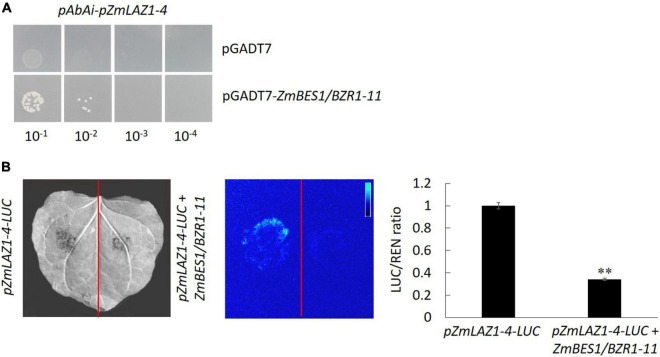
Confirmation of ZmBES1/BZR1-11 binding to *ZmLAZ1-4* promoter. The Y1H **(A)** and dual-luciferase assay **(B)** were performed to verify the binding and negative regulation of *ZmLAZ1-4* by ZmBES1/BZR1-11 transcription factor. The tobacco leaves were co-infiltrated by *35S*-*ZmBES1/BZR1-11* and *pZmLAZ1-4-LUC*, incubated at 22°C and 14 light/10 dark for 3 days, visualized for LUC signal, and used to measure activity LUC and REN. **p < 0.01.

In order to explore the mechanism of ZmLAZ1-4 regulating Zn transport, the co-expression analysis was conducted. The results showed that there were 27 genes co-expressed with *ZmLAZ1-4* with correlation coefficient > 0.9 or < −0.9, and only *ZmBES1/BZR1-11* among these candidates encoded transcription factor and negatively co-expressed with *ZmLAZ1-4* (Supplementary Table 2). The result of RT-qPCR likewise showed that the expression of *ZmLAZ1-4* and *ZmBES1/BZR1-11* was significantly downregulated and upregulated by Zn deficiency (5 μM), respectively (Figure 6). It was predicted that there were six E-boxes (CAXXTG) of BES1/BZR1 binding element (Yin et al., 2005) during *ZmLAZ1-4* promoter by PlantCARE. Hence, the yeast one-hybrid (Y1H) was performed. As shown in Figure 7, on Leu dropout SD medium, the growth of Y1H gold strain co-transformed by empty prey vector pGADT7 and pAbAi-*pZmLAZ1-4* harboring six E-boxes elements of *ZmLAZ1-4* promoter was inhibited by 200 ng/ml AbA, whereas the Y1H gold strain co-transformed by pGADT7*-ZmBES1/BZR1-11* and pAbAi-*pZmLAZ1-4* formed few colonies, indicating that the ZmBES1/BZR1-11 transcription factor could bind to *ZmLAZ1-4* promoter. The result was further verified by dual-luciferase assay *in vivo*. The relative LUC activity (LUC/REN) of leaves co-infiltrated by reporter vector *ZmLAZ1-4-LUC* and effector vector 35S-*ZmBES1/BZR1-11* was significantly lower than that of control (Figure 7B). These results indicate that the ZmBES1/BZR1-11 transcription factor binds to *ZmLAZ1-4* promoter to inhibit *ZmLAZ1-4* transcription.

## Discussion

The eight ZmLAZ1 members were grouped into a family in phylogenetic analysis because of their sequence similarity, especially their conserved DUF300 domain (Malinovsky et al., 2010; Liu et al., 2020). In the present study, only ZmLAZ1-4 and ZmLAZ1-8 were predicted to combine metal ions including Zn^2+^, Mg^2+^, or Ca^2+^ (Table 1). During prokaryotic expression, ZmLAZ1-8 was not successfully purified (Supplementary Figure 1). Eukaryotic membrane proteins are often difficult to be purified (Newstead et al., 2007). Therefore, the combination of ZmLAZ1-4 to predicted substrates was verified by thermal shift assay (Figure 1). Even in ZIP family, only some members were identified as Zn transporters (Grotz et al., 1998; Ishimaru et al., 2007; Evens et al., 2017; Yang et al., 2020). The other members might function as transporters of other divalent ions (Milner et al., 2013). The overexpression of *ZmLAZ1-4* in yeast mutant, *Arabidopsis*, and maize significantly increased Zn uptake (Figures 2–4), suggesting that the ZmLAZ1-4 protein was involved in Zn uptake in maize.

In our previous study, ZmLAZ1-4 was predicted to localize on chloroplast, plasmalemma, cytoplasm, and endoplasmic reticulum (Liu et al., 2020). The subcellular localization showed that ZmLAZ1-4 functioned on plasma and vacuolar membrane, as well as chloroplast using tonoplast maker AtTIP2 and plasma membrane marker OsRac3 (Figure 5 and Supplementary Figure 7), which well confirmed the tonoplast and plasma membrane localization (Loque et al., 2005; Tao et al., 2021). Hence, they could be used as a marker in our study. The phenomenon was similar with Mg^2+^ transporter AtMRS2 showing different intracellular localization patterns in yeast and chloroplast localization, and Pi transporter PHT2;1 localizing to mitochondria, plasma membrane, endoplasmic reticulum, and chloroplast (Wayne and Maria, 2002; Drummond et al., 2006). In *Arabidopsis*, two LAZ1 proteins were also localized on plasma and vacuolar membrane, but no specific marker was used for chloroplast localization (Malinovsky et al., 2010). Our result suggests that ZmLAZ1-4 functions on the plasma membrane and uptakes Zn from the soil, and transports Zn into vacuole. But the mechanism of ZmLAZ1-4 acting on chloroplast remains unclear. Before this study, Zn transport across chloroplast and vacuolar membrane was well documented to be mediated by HMA, MTP, and *Oryza sativa* Zn transporter (OTZ) proteins (Kobae et al., 2004; Arrivault et al., 2006; Martinoia et al., 2007; Moreno et al., 2008; Kim et al., 2009; Morel et al., 2009; Lan et al., 2013; Menguer et al., 2013; Tanaka et al., 2015). Some endoplasmic reticulum-localized and Golgi apparatus-localized zinc transporters were also involved in Zn homeostasis by controlling the release of zinc into cytosol (Fujiwara et al., 2015; Adulcikas et al., 2018; Wang et al., 2021). Our result of subcellular localization could not rule out the possibility of endoplasmic reticulum and Golgi apparatus localization of ZmLAZ1-4 (Supplementary Figure 7). This will be explored in further study.

**FIGURE 8:**
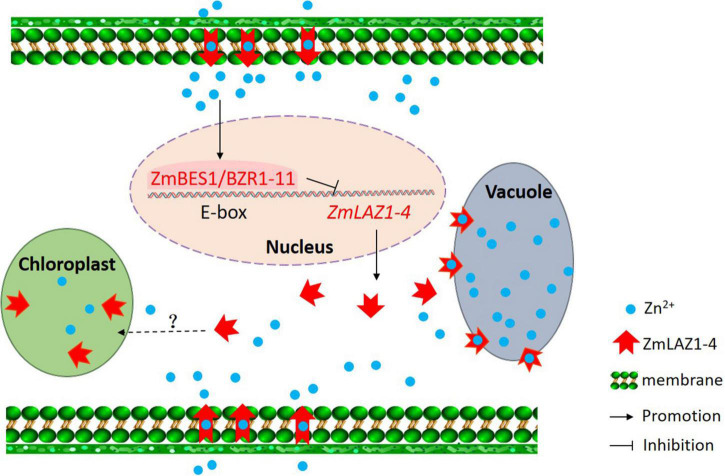
Signaling diagram of zinc homeostasis in maize. The black words, lines, and arrows indicate signaling pathways previously reported. The red words, lines, and arrows show signaling pathway demonstrated in the study.

Among the 27 genes co-expressing with *ZmLAZ1-4*, only *ZmBES1/BZR1-11* encoded transcription factor and negatively co-expressed with *ZmLAZ1-4* (Figure 6 and Supplementary Table 2). The ZmBES1/BZR1-11 can bind to E-boxes (CAXXTG) element in *ZmLAZ1-4* promoter to inhibit *ZmLAZ1-4* transcription (Yin et al., 2005), which was verified by Y1H and dual-luciferase assay (Figure 7). But previous studies exhibited that BES1/BZR1 transcription factor responds to BR induction and regulates the expression of BR-responsive genes (Yin et al., 2005; Yu et al., 2018), and *Arabidopsis* LAZ1 proteins localized on plasma and vacuolar membrane also mediated BR signaling (Malinovsky et al., 2010). It could be concluded that the ZmLAZ1-4 protein functioned as a Zn^2+^ transporter on plasma and vacuolar membrane, and chloroplast to modulate Zn homeostasis in maize. The expression of *ZmLAZ1-4* was negatively regulated by ZmBES1/BZR1-11 transcription factor. The results of this study indicated that ZmLAZ1-4 was a novel zinc transporter distinct from the previously documented Zn transporters ZIP, ZRT, IRT, NRAMP, etc. (Grotz et al., 1998; Ishimaru et al., 2007; Milner et al., 2013; Evens et al., 2017; Yang et al., 2020), as plotted in a signaling diagram of zinc homeostasis together with the previously reported evidence (Figure 8).

## Conclusion

The ZmLAZ1-4 protein is a novel zinc transporter that transports zinc ions across plasma and vacuolar membrane and modulates zinc homeostasis under the negative regulation of ZmBES1/BZR1-11 transcription factor.

## Data Availability Statement

Publicly available datasets were analyzed in this study. These data can be found here: wap.maizegdb.org, Zea_mays.AGPv4.32gff3.

## Author Contributions

FF and WL conceived and supervised the research. BL, HY, QyY, LD, FS, JQ, WF, and QqY performed the experiments. BL and WL drafted the manuscript. BL and HY revised the manuscript. All authors interpreted and discussed the data.

## Conflict of Interest

The authors declare that the research was conducted in the absence of any commercial or financial relationships that could be construed as a potential conflict of interest.

## Publisher’s Note

All claims expressed in this article are solely those of the authors and do not necessarily represent those of their affiliated organizations, or those of the publisher, the editors and the reviewers. Any product that may be evaluated in this article, or claim that may be made by its manufacturer, is not guaranteed or endorsed by the publisher.

## Footnotes

1
https://swissmodel.expasy.org/

2
https://www.rcsb.org/

3
http://www.cbs.dtu.dk/services/TMHMM

4
https://www.maizegdb.org

5
http://bioinformatics.psb.ugent.be/webtools/plantcare/html/
