# Supplementary material for: Zinc Transporter ZmLAZ1-4 Modulates Zinc Homeostasis on Plasma and Vacuolar Membrane in Maize
Source: Front Plant Sci. 2022 May 2;13:881055. doi: 10.3389/fpls.2022.881055 (PMC9108671; doi:10.3389/fpls.2022.881055)
Supplement: Supplementary file 1 [file Data_Sheet_1.zip › Supplementary Dataset 1.docx]

**Supplementary Data Set S1** A Perl script for maize gene common expression

use warnings;

use strict;

my $usage = "Usage: perl $0 gene_name\nNote: gene_name is Maize_AGPv3_gene.\n";

my $query = $ARGV[0];

if ($query =~ /-h/) {

die "$usage";

}

open IN,"./data/Dataset S1.txt";

open OUT,">$query-com.txt";

my $title = <IN>;

my @title = split/\s+/,$title,2;

print OUT "NO\t$title[0]\t"."r\t$title[1]";

my %data = ();

while (<IN>) {

chomp;

my @a = split/\s+/,$_,2;

$data{$a[0]} = [split/\s+/,$a[1]];

}

close IN;

my @result = ();

foreach my $key (keys %data) {

my @a = @{$data{$query}};

my @b = @{$data{$key}};

my @num = ();

my $x;

my $y;

my $c;

my $x2;

my $y2;

foreach (3..$#a) {

if ($a[$_] =~ /\d+/ && $b[$_] =~ /\d+/) {

push @num,$_;

$x += $a[$_];

$y += $b[$_];

}

}

next if $#num+1 < 1;

$x = $x/($#num+1);

$y = $y/($#num+1);

foreach (@num) {

$c += (($a[$_]-$x)*($b[$_]-$y));

$x2 += (($a[$_]-$x)*($a[$_]-$x));

$y2 += (($b[$_]-$y)*($b[$_]-$y));

}

next if (sqrt($x2)*sqrt($y2)) == 0;

my $r = $c/(sqrt($x2)*sqrt($y2));

my $tmp;

foreach (0..$#{$data{$key}}) {

$tmp .= "\t${$data{$key}}[$_]";

}

push @result,"$key\t$r$tmp";

}

my $cnt = 1;

foreach (sort{abs($b->[1])<=>abs($a->[1])}map{[split/\s+/,$_,3]}@result) {

print OUT "$cnt\t$_->[0]\t$_->[1]\t$_->[2]\n";

$cnt++;

}

close OUT;
