# Supplementary material for: Zinc Transporter ZmLAZ1-4 Modulates Zinc Homeostasis on Plasma and Vacuolar Membrane in Maize
Source: Front Plant Sci. 2022 May 2;13:881055. doi: 10.3389/fpls.2022.881055 (PMC9108671; doi:10.3389/fpls.2022.881055)
Supplement: Supplementary file 1 [file Data_Sheet_1.zip › Supplementary Figures.docx]

**Supplementary Figure 1** SDS detection of purified ZmLA1-4 and ZmLA1-8 from their prokaryotic expression stains.


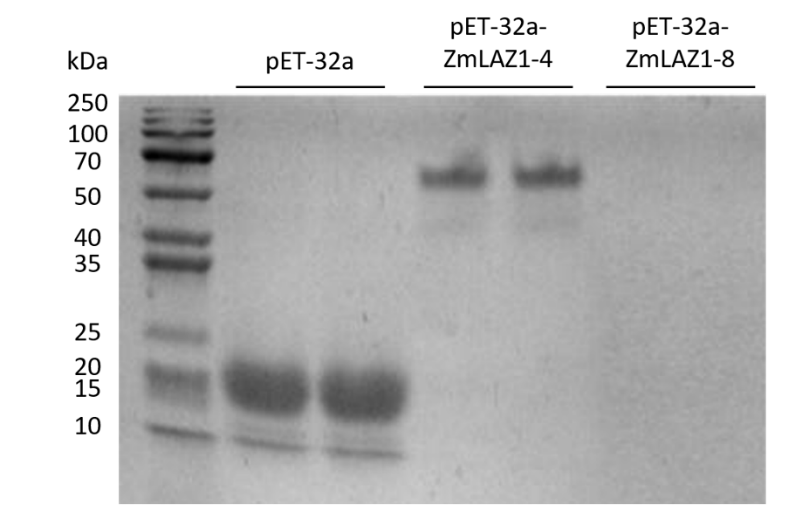


**Supplementary Figure 2** PCR identification of homozygous T_3_ *Arabidopsis* lines.


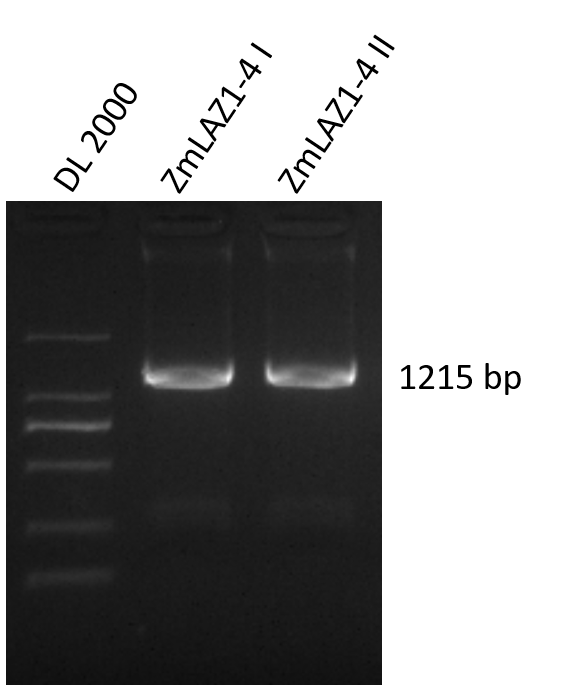


**Supplementary Figure 3** Screening of regenerated plantlets by PAT/bar EPSPS LFD Strips. The red arrow indicates that the target gene has been detected and this strain is positive.


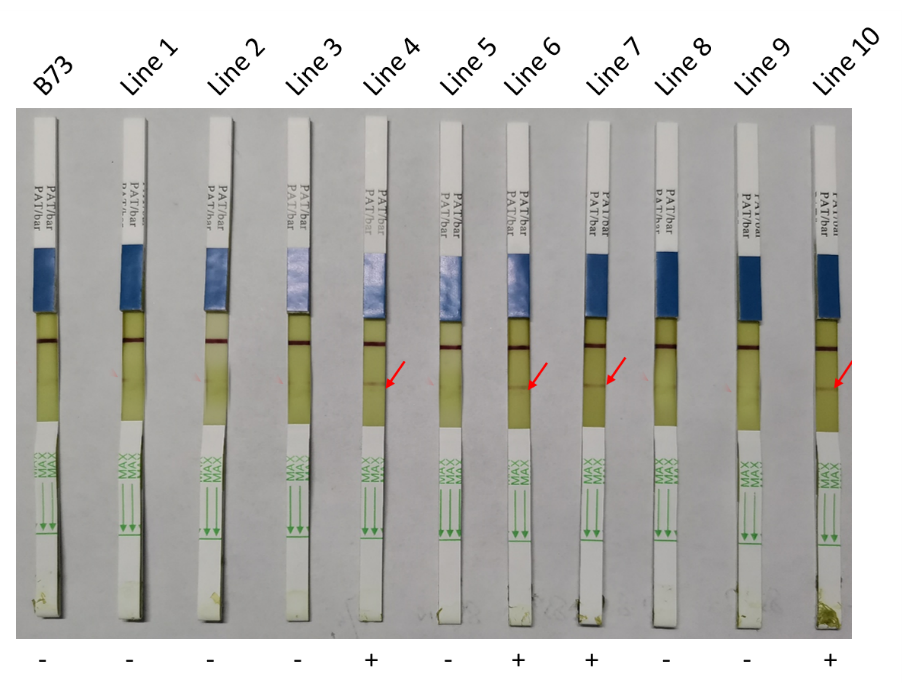


**Supplementary Figure 4** PCR identification of homozygous T_3_ maize lines.


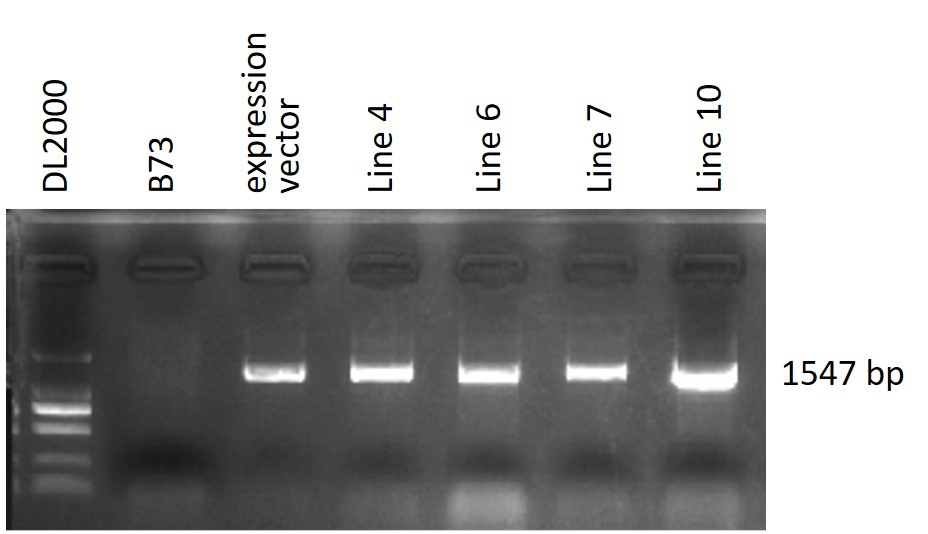


**Supplementary Figure 5** Relative expression level of *ZmLA1-4* gene in homozygous T_3_ lines. The values were presented as the mean ± standard deviation of three replicates. ** P < 0.01.

**
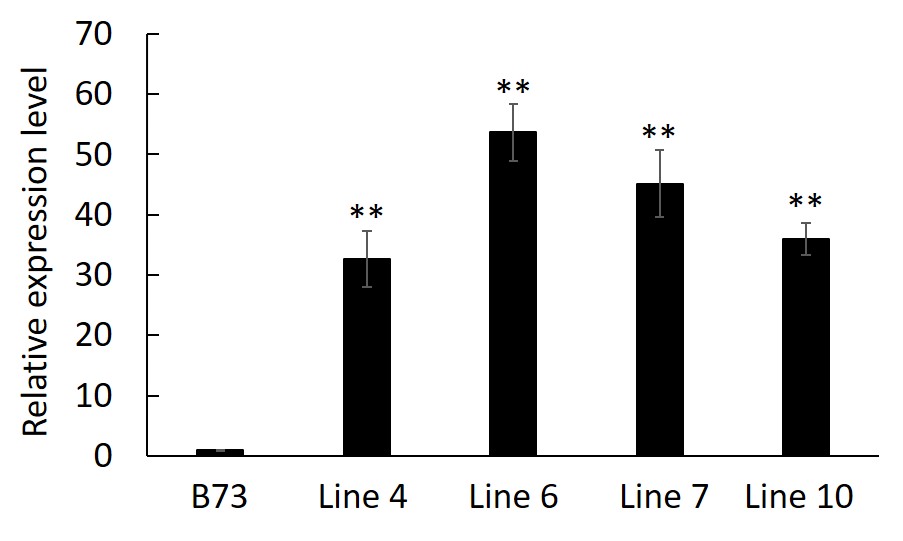
**

**Supplementary Figure 6** Predicted transmembrane domains of ZmLAZ1-4 by TMHMM.


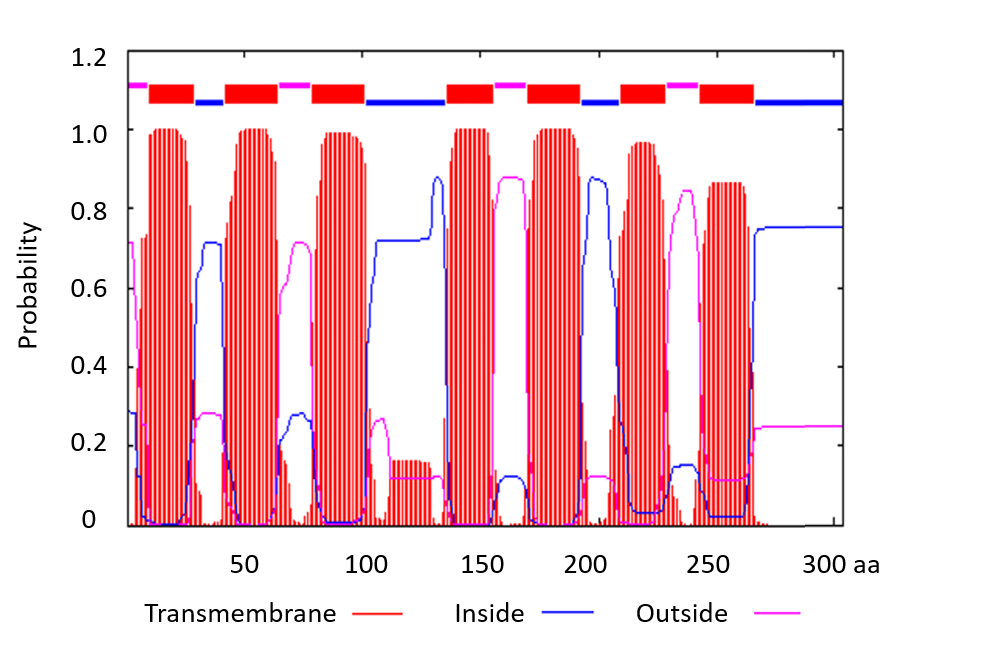


**Supplementary Figure 7** Subcellular localization of ZmLAZ1-4 in leaves of *Nicotiana benthamiana*.

**
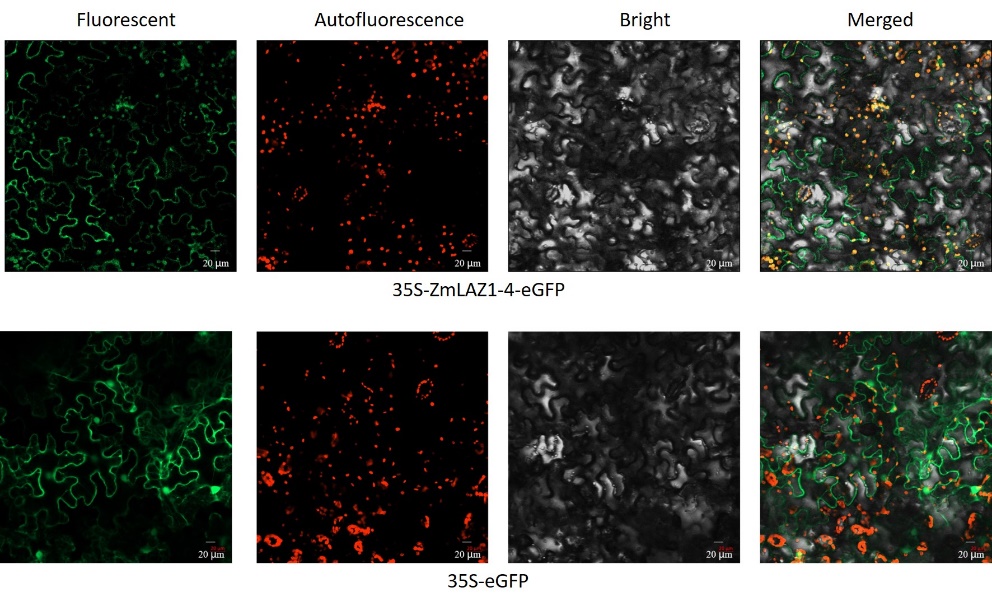
**
