# Supplementary material for: Zinc Transporter ZmLAZ1-4 Modulates Zinc Homeostasis on Plasma and Vacuolar Membrane in Maize
Source: Front Plant Sci. 2022 May 2;13:881055. doi: 10.3389/fpls.2022.881055 (PMC9108671; doi:10.3389/fpls.2022.881055)
Supplement: Supplementary file 1 [file Data_Sheet_1.zip › Supplementary Table 1.docx]

**Supplementary Table S1 PCR primers used in this study**

| Sequence | Primer | Sequence | Amplified length | Usage |
| --- | --- | --- | --- | --- |
| CDS of *ZmLAZ1-4* | CDS1-4F | 5'-cgagctccgtcgacaagcttATGGAGTGGGCGTCGGCGGCGT-3' | 1255 bp | Construction of prokaryoutic expression vector pET32a-*ZmLA1-4*. |
|  | CDS1-4R | 5'-tggtggtggtggtgctcgagTCATTGTTTTCTTACATCGGTG-3' |  |  |
| CDS of *ZmLAZ1-8* | CDS1-8F | 5'-cgagctccgtcgacaagcttATGGAGTGGGCGTCGGCGGCGT-3' | 1483 bp | Construction of prokaryoutic expression vector pET32a-*ZmLA1-8*. |
|  | CDS1-8R | 5'-tggtggtggtggtgctcgagTCATTGTTTTCTTACATCGGTG-3' |  |  |
| CDS of *ZmLAZ1-4* | pYES2F | 5’-ttggtaccgagctcggatccATGGAGTGGGCGTCGGCGGCGT-3’ | 1225 bp | Construction of yeast expression vector pYES-*ZmLAZ-4*. |
|  | pYES2R | 5’-gatggatatctgcagaattcTCATTGTTTTCTTACATCGGTG-3’ |  |  |
| Fragment of *ZmLAZ1-4* | LAZ4F1 | 5’-ATGGCGGTGGAGTTGGCCAAGC-3’ | 1215 bp | PCR identification of *Arabidopsis* transgenic lines. |
|  | LAZ4R1 | 5’-GCTAGTAATCCATCTGTTCCCT-3’ |  |  |
| Fragment of *ZmLAZ1-4* | LAZ4F2 | 5’-CGCCCGCCGTAATAAATAGA-3’ | 1547 bp | PCR identification of maize transgenic lines. |
|  | LAZ4R2 | 5’-TGTTCGGCCACTCAAACTTA-3’ |  |  |
| Fragment of *ZmLAZ1-4* | LAZ4F3 | 5'-CTAATAGCAGCCGTTGGGCA-3' | 287 bp | RT-qPCR analysis |
|  | LAZ4R3 | 5'-ACTGGACTGTTTGAGGCCG-3' |  |  |
| Fragment of *ZmGAPDH* | GAPDHF | 5’-CCATCACTGCCACACAGAAAAC-3’ | 170 bp |  |
|  | GAPDHR | 5’-AGGAACACGGAAGGACATACCAG-3’ |  |  |
| Fragment of *ZmBES1/BZR1-11* | BES1F | 5'-AACGAAATGCCCAACGGTAT-3' | 153 bp |  |
|  | BES1R | 5'-CGAGCTTAGAGGAGGGGTTA-3' |  |  |
| CDS of *ZmLAZ1-4* without stop codon | NonTerm1-4F  NonTerm1-4R | 5'-gagaggacagggtaccATGGCGGTGGAGTTGGCCAAGC-3' | 1252 bp | Construction of expression vector pC2300-*35S-ZmLAZ1-4-eGFP*. |
|  |  | 5'-accatggtactagtgtcgacGCTAGTAATCCATCTGTTCCCT-3' |  |  |
| CDS of *AtTIP2* without stop codon | AtTIP2F  AtTIP2R | 5'-GCTTAAATCTCAGCCGTCCG-3' | 756 bp | Construction of expression vector mCherry-*AtTIP2-eGFP*. |
|  |  | 5'-GCAATGGCCACTCTTGAAACC-3' |  |  |
| CDS of *ZmBES1/BZR1-11* | pGADT7F | 5’-accagattacgctcatatgATGGCGAGCGGCGGCGGCGGGG-3’ | 1035 bp | Construction of Y1H vector pGADT7-*ZmBES1/BZR1-11* |
|  | pGADT7R | 5’-tgcccacccgggtggaattcCTAGAGCTTCAGGGTCAGCTCC-3’ |  |  |
| Promoter sequence of *ZmLA1-4* | pAbAiF | 5’-tcgagctcggtacccgggGAGGTATTGAGTGATTTCTTGT-3’ | 1100 bp | Construction of Y1H prey vector pGADT7-*ZmBES1/BZR1-11*. |
|  | pAbAiR | 5’-gcacatgcctcgaggtcgacCTTGGGCAAACGGAGAGGGGTT-3’ |  |  |
| Fragment of *ura3-52* | Y1HF | 5’-GGATGAGTAGCAGCACGTTCCTTATATGTAGCTTTC-3’ | 1815 bp | PCR identification of positive yeast clones. |
|  | Y1HR | 5’-GGCAGTTTGGAGGTCTCTCTGATAGAAACC-3’ |  |  |
| Promoter sequence of *ZmLA1-4* | pGreenIIF | 5’-tcgacggtatcgataagcttGAGGTATTGAGTGATTTCTTGT-3’ | 1100 bp | Construction of reporter vector p*ZmLAZ1-4-LUC* |
|  | pGreenIIR | 5’-gctctagaactagtggatccCTTGGGCAAACGGAGAGGGGTT-3’ |  |  |
| CDS of *ZmBES1/BZR1-11* | pCAMBIA2300F | 5’-cacgggggacgagctcATGGCGAGCGGCGGCGGCGGGG-3’ | 1075 bp | Construction of effector vector pCAMBIA2300-*35S-ZmBES1/BZR1-11-eGFP* |
|  | pCAMBIA2300R | 5’-tgctcaccatggtaccGAGCTTCAGGGTCAGCTCCAGG-3’ |  |  |
