# Supplementary material for: Zinc Transporter ZmLAZ1-4 Modulates Zinc Homeostasis on Plasma and Vacuolar Membrane in Maize
Source: Front Plant Sci. 2022 May 2;13:881055. doi: 10.3389/fpls.2022.881055 (PMC9108671; doi:10.3389/fpls.2022.881055)
Supplement: Supplementary file 1 [file Data_Sheet_1.zip › Supplementary Table 2.docx]

**Supplementary Table S2 Co-expressing genes with *ZmLAZ1-4***

| Accession number | Correlation coefficient | Protein description |
| --- | --- | --- |
| GRMZM2G052403 | 0.93 | AAA-type ATPase family protein |
| GRMZM2G179662 | -0.93 | Protein ABHD17C serine/threonine-protein kinase, VPS15 phosphoinositide 3-kinase regulatory subunit 4, protein kinase family protein / WD-40 repeat family protein |
| GRMZM2G145065 | 0.93 | Uncharacterized |
| GRMZM2G036169 | 0.93 | Uncharacterized |
| GRMZM2G025074 | -0.93 | Uncharacterized |
| GRMZM6G287292 | -0.93 | ZmBES1/BZR1-11 |
| GRMZM2G164308 | -0.93 | ABA-induced protein uncharacterized |
| GRMZM2G122061 | 0.92 | AAA-type ATPase family protein uncharacterized |
| GRMZM2G051792 | 0.92 | Probable E3 ubiquitin ligase SUD1 |
| GRMZM2G3759041 | -0.92 | Serine/threonine-protein kinase tricorner-like putative AGC protein kinase family protein |
| GRMZM2G411536 | 0.91 | E3 ubiquitin-protein ligase UPL1 |
| GRMZM2G045999 | -0.91 | Plasma membrane |
| GRMZM2G148411 | 0.91 | TLD-domain containing nucleolar protein |
| GRMZM2G133631 | 0.91 | Abscisic acid receptor PYL9 |
| GRMZM5G876773 | -0.91 | Plasma membrane |
| GRMZM2G038412 | -0.91 | AT4g33690/T16L1_180-like protein |
| GRMZM2G432566 | -0.91 | RING/U-box superfamily protein putative RING zinc finger domain superfamily protein |
| GRMZM2G020553 | 0.91 | Phospholipid-transporting ATPase 3 |
| GRMZM2G431900 | -0.90 | Charged multivesicular body protein 2a |
| GRMZM2G091433 | -0.90 | CCAAT-HAP5-transcription factor 59 repressor protein subunit of Dr1/DrAp1 repressor complex |
| GRMZM2G118363 | 0.90 | TOM1-like protein 6 ADP-ribosylation factor-binding protein GGA3 ENTH/VHS/GAT family protein |
| GRMZM2G167520 | 0.90 | Brittle stalk-2-like protein 6 Protein COBRA |
| GRMZM2G104769 | 0.90 | E3 ubiquitin-protein ligase CHIP STIP1 y and U box-containing protein 1 |
| GRMZM2G144440 | 0.90 | Aspartic proteinase A1 |
| GRMZM2G011800 | -0.90 | WPP domain-associated protein |
| GRMZM2G006477 | -0.90 | Putative MYB DNA-binding domain superfamily protein |
| GRMZM2G442195 | 0.90 | Uncharacterized |
